# Astragaloside IV suppresses neuroinflammation via PI3K/Akt/NF-κB to ameliorate cerebral ischemia-reperfusion injury based on network pharmacology analysis and experimental validation

**DOI:** 10.3389/fimmu.2026.1735000

**Published:** 2026-04-02

**Authors:** Xiaoxuan Wu, Nan Guo, Xiaofen Zhang, Lingjun Kong, Jing Zhang, Baokai Dou

**Affiliations:** Department of Pharmacy, Shandong Provincial Hospital Affiliated to Shandong First Medical University, Jinan, Shandong, China

**Keywords:** astragaloside IV, cerebral ischemia-reperfusion injury, network pharmacology, neuroinflammation, PI3K/Akt/NF-κB signaling pathway

## Abstract

**Aim:**

This study aimed to systematically investigate the therapeutic effects and underlying mechanisms of ASIV against cerebral ischemia-reperfusion injury (CIRI) using an integrated approach combining network pharmacology and experimental validation.

**Methods:**

Potential targets of Astragaloside IV (ASIV) were predicted using SwissTargetPrediction, PharmMapper and Comparative Toxicogenomics Database (CTD). Ischemic stroke-related targets were collected from GeneCards, DisGeNET and DrugBank. Overlapping targets were used to construct a protein-protein interaction (PPI) network via STRING and visualized in Cytoscape. Functional enrichment analysis of Gene Ontology (GO) and Kyoto Encyclopedia of Genes and Genomes (KEGG) pathways was performed using DAVID. The neuroprotective effects of ASIV were evaluated in a mouse model of middle cerebral artery occlusion/reperfusion (MCAO/R), assessing neurological function, infarct volume, blood brain barrier (BBB) integrity, inflammatory markers, and PI3K/Akt/NF-κB pathway activity. *In vitro*, LPS-stimulated BV-2 microglia were used to examine the effects of ASIV on cell viability, inflammatory cytokine expression, and PI3K/Akt/NF-κB signaling.

**Results:**

Network pharmacology analysis identified 166 overlapping targets, and enrichment analysis emphasized the PI3K/Akt pathway as a key mechanism. In MCAO/R mice, ASIV significantly improved neurological function, reduced infarct volume and neuronal apoptosis, decreased Evans blue leakage, attenuated MMP-9 expression, and restored ZO-1 and Occludin levels. ASIV also suppressed the mRNA levels of IL-6, IL-1β, and TNF-α both *in vivo* and in LPS-stimulated BV-2 cells. Additionally, ASIV effectively upregulated the expression of Anti-inflammatory cytokines (IL-10 and Arg-1) in LPS-induced BV2 cells. Mechanistically, ASIV suppresses NF-κB activation through the stimulation of the PI3K/Akt signaling pathway.

**Conclusion:**

ASIV exerts neuroprotective effects against CIRI by inhibiting neuroinflammation and preserving BBB integrity likely through modulation of the PI3K/Akt/NF-κB signaling pathway.

## 1 Introduction

Ischemia stroke represents a leading cause of mortality and long-term disability worldwide (1). Epidemiological data from 2021 revealed that ischemic stroke accounted for 69.9 million prevalent cases, 7.8 million incident cases, 3.6 million deaths, and a loss of 70.4 million disability-adjusted life years (DALYs) globally. Notably, the proportion among all strokes types has risen from 68.32% in 1990 to 73.33%, cementing its role as the dominant cerebrovascular disease (2). Current standard treatment focuses on rapid revascularization through thrombolysis agents or mechanical thrombectomy, is the cornerstone of rapid revascularization. However, the restoration of blood flow can paradoxically trigger a cascade of secondary pathological events collectively termed CIRI (3, 4).

The pathophysiology of CIRI is multifaceted; a key contributor to CIRI is neuroinflammation, which involves the activation of microglia, disruption of the BBB, and release of pro-inflammatory cytokines, collectively amplifying ischemic tissue injury (5–7). Despite advancements in acute stroke management, effective therapeutic strategies specifically designed to mitigate CIRI and counterbalance its inflammatory components remain limited, highlighting the urgent need to develop novel neuroprotective agents that can simultaneously target multifactorial mechanisms/pathways.

Astragaloside IV (ASIV), a principal bioactive component derived from the traditional Chinese herb *Astragalus membranaceus*, has emerged increasing attention for its promising neuroprotective potential in preclinical studies of ischemic stroke (8, 9). Accumulating studies have demonstrated that ASIV exerts therapeutic effects in multiple aspects, including anti-inflammatory, antioxidant, and anti-apoptotic activities, along with promoting angiogenesis (9, 10). Our previous research further revealed its immunomodulatory capabilities, showing that ASIV confers significant cerebroprotection in a MCAO/R model by specifically inhibiting the post-ischemic brain infiltration of Natural Killer (NK) cells (11, 12). However, the encouraging evidence is mainly based on animal studies, ASIV remains a promising multi-target candidate that requires further research to advance its clinical translation for adjuvant stroke therapy. Network pharmacology, a systems biology approach that integrates drug-target-disease interactions, has emerged as a powerful tool to uncover the multi-target mechanisms of traditional Chinese medicine compounds (13, 14). The multi-factorial and complex nature of ischemic brain injury suggests that compounds like ASIV may act through multiple targets, thus requiring a comprehensive and systematic research approach.

In the present study, we systematically investigated the therapeutic effects of ASIV and its underlying mechanisms against CIRI by integrating network pharmacology with subsequent *in vivo* and *in vitro* experiments. Our network pharmacology analysis suggested that the PI3K/Akt signaling pathway might be a central potential pathway for the action of ASIV. Guided by these predictions, we conducted experimental verification using a mouse MCAO/R model and LPS-induced BV-2 microglia experiments. Our research results show that ASIV could significantly improve neurological function, reduce cerebral infarction volume, maintain the integrity of the BBB, and alleviates neuroinflammation.

Importantly, we confirmed that at least part of these protective effects are mediated by activating the PI3K/Akt pathway and subsequently regulating the NF-κB signaling axis. These findings not only provide new mechanistic insights into the role of ASIV but also offer a solid experimental foundation for its potential development as an adjunctive treatment for ischemic stroke.

## 2 Article types

Original research.

## 3 Methods

### 3.1 Network pharmacological analysis

The canonical SMILES notation and 3D structural data of ASIV were retrieved from the PubChem database (https://pubchem.ncbi.nlm.nih.gov/). The predicted targets of ASIV were collected from SwissTargetPrediction (http://swisstargetprediction.ch/), PharmMapper (https://www.lilab-ecust.cn/pharmmapper/), and CTD (https://ctdbase.org/). “Cerebral ischemia”, “brain ischemia” and “ischemic stroke” were input into GeneCards (https://www.genecards.org/), DisGeNET (https://disgenet.com/), and DrugBank (https://go.drugbank.com/) as keywords to gather ischemic stroke associated target genes. Potential targets of ASIV and ischemic stroke associated targets were analyzed using Venny 2.1.0 (https://bioinfogp.cnb.csic.es/tools/venny/) to identify overlapping targets, and the results are represented in a Wayne diagram.

The overlapping targets were analyzed using the STRING database (https://string-db.org/) with species restricted to “Homo sapiens” and confidence ratings 0.900 to construct a PPI network. The network file (TSV format) was imported into Cytoscape 3.7.2 software (https://cytoscape.org/), and core targets were selected using Network Analyzer based on the degree value. In addition, the overlapping targets were imported into the DAVID database (https://davidbioinformatics.nih.gov/), and the analyzed organisms were selected as “Homo sapiens” for GO and KEGG pathway enrichment analysis. Finally, the results were visualized through the microbial system platform (http://www.bioinformatics.com.cn).

### 3.2 MCAO/R surgery and drug treatment

All animal experiments were performed under the approval of the Guide for the Care and Use of Laboratory Animals and the protocols were approved by Animal Ethical Committee of Shandong Provincial Hospital Affiliated to Shandong First Medical University (NSFC: NO.2023-012). Male C57BL/6 mice (8-week-old, 21–25 g) purchased from Beijing Vital River Laboratory Animal Technology Co., Ltd. underwent right MCAO under 1.5% halothane inhalation anesthesia for 45 min, after which the silicone-covered sutures were removed to initiate reperfusion as previously described (15). MCAO/R mice were randomly divided into sham, MCAO/R, and MCAO/R + ASIV groups. ASIV (purity >98%, Tongtian Biotechnology, Shanghai, China) was delivered at a dose of 40 mg/kg through intraperitoneal injection immediately after reperfusion and 12 h later (11, 12). For euthanasia, mice were deeply anesthetized with an intraperitoneal injection of pentobarbital sodium (50 mg/kg). After the disappearance of the hindlimb pedal withdrawal reflex, the brain was collected either by cervical dislocation or by transcardiac perfusion with phosphate-buffered saline (PBS) to remove blood, depending on experimental requirements (16–18).

### 3.3 Neurological deficit scoring

Neurological deficits were assessed at 24 h post-reperfusion by investigators blinded to the treatment conditions using a modified 12-point neurological scoring system as previously described (19).

### 3.4 Infarct volume measurement and brain edema

After 24 hours of reperfusion, the mice were anesthetized with pentobarbital sodium (50 mg/kg, i.p.) and then euthanized by cervical dislocation. The brains were immediately removed for TTC staining to assess the infarct volume. Briefly, six 1-mm coronal brain sections were stained with 2% TTC (2, 3,5-triphenyl tetrazolium chloride, Sigma, USA) at 37 °C for 15 minutes, and fixed in 4% paraformaldehyde. Infarct areas were analyzed using ImageJ software, with total volume calculated by summing the infarct areas across sections and multiplying by slice thickness. Ipsilateral hemispheric edema was quantified as the difference in total area between the ipsilateral and contralateral hemispheres (20).

### 3.5 Immunofluorescence staining and TUNEL staining

Brain sections were incubated overnight at 4 °C with rabbit anti-NeuN (1:500, Abcam) and rabbit anti-Iba-1 (1:500, Abcam). After washing, the sections were then incubated at room temperature for 2 hours with Alexa Fluor 594-conjugated goat anti-rabbit antibody (1:1000, Life Technologies) and Alexa Fluor 488-conjugated goat anti-rabbit antibody (1:1000, Life Technologies), respectively. Nuclei were stained with DAPI, and immunolabeled signals were captured using a Leica SP8 confocal microscope. Quantitative analysis of the results was performed using ImageJ.

To further evaluate the effect of ASIV on neuronal apoptosis, dual staining was performed on brain sections using the neuronal marker NeuN and the TUNEL BrightGreen Apoptosis Detection Kit (Vazyme). TUNEL staining was carried out strictly in accordance with the manufacturer’s instructions, and the number of TUNEL-positive neurons was manually counted in the ischemic cortical region under a fluorescence microscope.

### 3.6 Evans blue extravasation

BBB integrity was assessed through Evans blue extravasation. MCAO/R mice received 2% Evans blue (Sigma-Aldrich, USA) in saline (4 mL/kg) via tail vein injection at the onset of reperfusion. After 24 h, the mice were anesthetized by an intraperitoneal injection of sodium pentobarbital (50 mg/kg, i.p.) and immediately subjected to cardiac perfusion with cold PBS. Ipsilateral hemispheres were dissected, weighed, and homogenized in 400 μL of 50% trichloroacetic acid. Following centrifugation (12,000 ×g, 40 min), Evans blue concentration in supernatants was quantified spectrophotometrically at 620 nm. The results were calculated against a standard curve and expressed as μg/g tissue.

### 3.7 Real-time PCR

Total RNA was extracted using Trizol reagent (Invitrogen, Carlsbad, CA) following the manufacturers’ instructions. The ReverTra Ace qPCR Kit was used to reverse transcribe RNA into cDNA. The SYBR Green PCR kit (Toybo, Japan) was used to test the mRNA levels according to gene-specific primers. The primer sequences are shown in **Table 1**. The mRNA levels were normalized against β-actin and presented as 2^–ΔΔCT^.

**Table 1:** List of primers used for RT-qPCR.

Species	Gene	Forward primer(5′-3′)	Reverse primer(5′-3′)
Mouse	MMP-2	CAGGGCACCTCCTACAACAG	CAGTGGACATAGCGGTCTCG
Mouse	MMP-9	TAGATCATTCCAGCGTGCCG	GCTTAGAGCCACGACCATACA
Mouse	IL-1β	TGTGTTTTCCTCCTTGCCTCTGAT	TGCTGCCTAATGTCCCCTTGAAT
Mouse	IL-6	TCACAGAAGGAGTGGCTAAGGACC	ACGCACTAGGTTTGCCGAGTAGAT
Mouse	TNF-α	TGAGCACAGAAAGCATGATCCG	GTAGACAGAAGAGCGTGGTGGC
Mouse	β-actin	TGTCCACCTTCCAGCAGAT	CTCAGTAACAGTCCGCCTAGA

### 3.8 Western blotting

Proteins were extracted from the ipsilateral cortex using RIPA buffer supplemented with protease and phosphatase inhibitors. The samples were separated by SDS-PAGE, transferred to PVDF membranes. The blots were blocked and incubated overnight at 4 °C with rabbit anti-ZO-1 (1:5000, Proteintech), rabbit anti-Occludin (1:5000, Proteintech), rabbit anti-PI3K (1:1000, Cell Signaling Technology), rabbit anti-p-PI3K (1:1000, Cell Signaling Technology), rabbit anti-Akt (1:1000, Abmart), rabbit anti-p-Akt (1:2000, Abmart), rabbit anti-phospho-NF-κB P65 (1:1000, Cell Signaling Technology), mouse anti-NF-κB P65 (1:1000, Cell Signaling Technology), mouse anti-β-actin (1:5000, Servicebio) or rabbit anti-GAPDH (1:5000, Proteintech). After the membranes were incubated with secondary antibodies for 2 hours at room temperature, protein bands were visualized using enhanced chemiluminescence.

### 3.9 Cell viability assay

The BV-2 cells were seeded into a 96-well plate at a density of 2 × 10^4^ cells/well and cultured at 37 °C with 5% CO_2_. After overnight attachment, cells were treated for 24 hours with various concentrations of ASIV (0, 5, 25, 50, 100, and 200 μM), either alone or in combination with LPS (100 ng/ml). The medium was then replaced with a fresh medium with 10 µL of CCK-8, and the cells were incubated for another 0.5 h. The absorbance was measured at 450 nm using a microplate reader (Infinite E plex, Tecan, Switzerland).

### 3.10 Statistical analysis

All data are presented as mean ± SEM. Statistical analysis was performed using GraphPad Prism10. In comparisons between groups, Student’s t-test was applied for two-group comparisons, while one-way ANOVA was used for multiple-group comparisons. Statistical significance was considered when the P value was less than 0.05.

## 4 Results

### 4.1 Network pharmacology analysis

**Figure 1:**
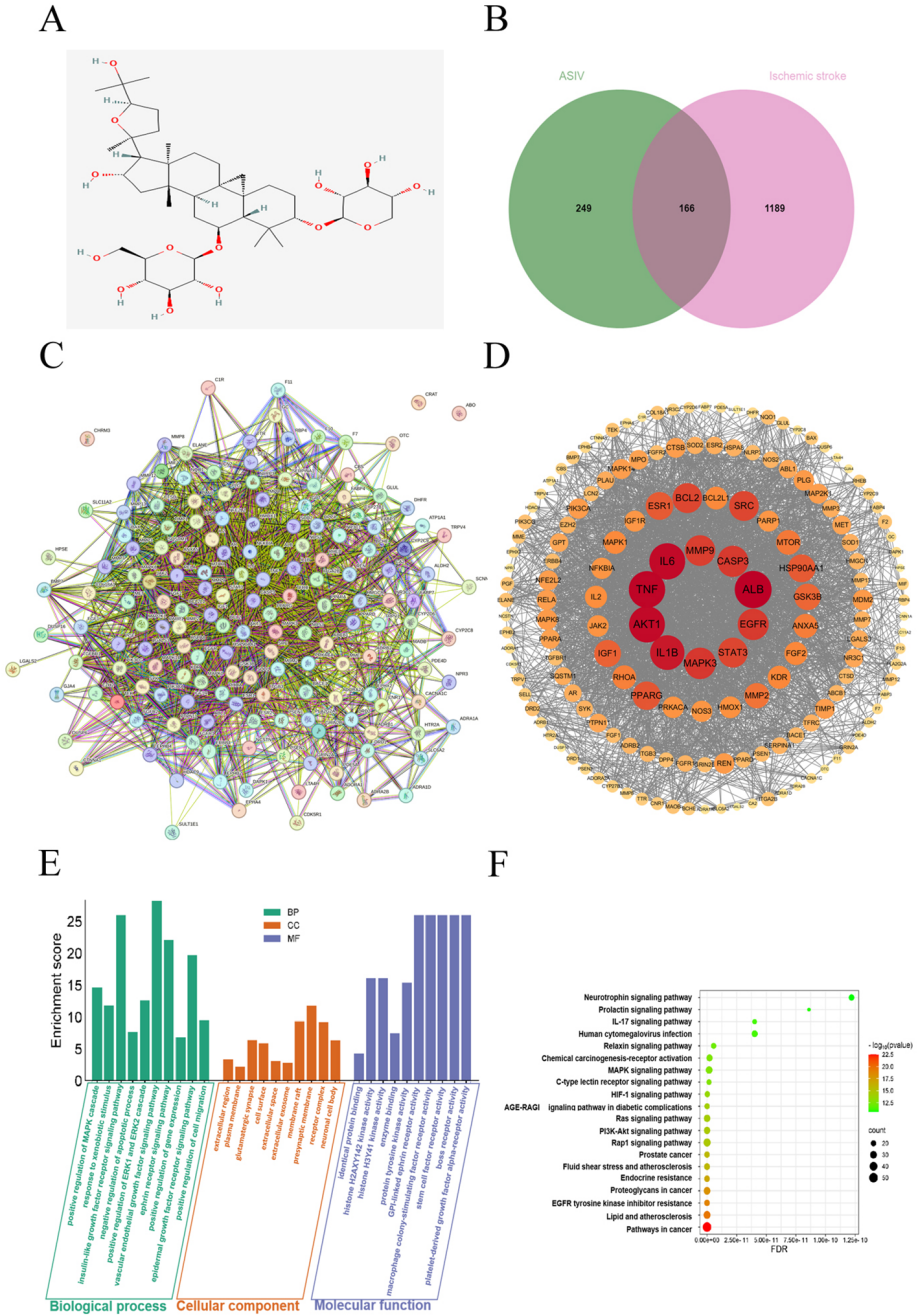
Network pharmacology analysis. **(A)** Chemical structure of ASIV. **(B)** Venn diagram of ASIV and ischemic stroke-associated targets. Includes 415 ASIV-related targets (left), 1355 ischemic stroke-related targets (right), and 166 overlapping targets (center). **(C)** PPI network of common targets generated by STRING. **(D)** The visualized PPI network in Cytoscape. After visualizing the nodes, bigger sizes and colors from to yellow red refer to higher degree values. **(E)** GO functional enrichment analysis. **(F)** KEGG pathway enrichment analysis.

Network pharmacology was used to explore the potential neuroprotective mechanisms of ASIV against cerebral ischemic injury. The chemical structure of ASIV was obtained from the PubChem database (**Figure 1A**). A total of 415 ASIV-related targets were identified from the SwissTargetPrediction, PharmMapper, and CTD databases. Additionally, 1,355 ischemic stroke-associated targets were retrieved from the GeneCards, DisGeNET, and DrugBank databases. Using VENNY 2.1.0 to generate a Venn diagram, 166 intersections were identified as potential candidate targets for ASIV in the treatment of ischemic stroke (**Figure 1B**). These intersection targets were uploaded to the STRING12.0 database to construct a PPI network (**Figure 1C**). The PPI network was visualized and analyzed using Cytoscape 3.7.2 software, and key genes were identified based on their degree centrality, including ALB, TNF, IL6, AKT1, IL1B, MAPK3, EGFR, CASP3, MMP9 and STAT3 (**Figure 1D**).

To clarify the potential functions of these targets, GO function and KEGG pathway enrichment analyses were performed on the 166 potential targets using the DAVID database. GO enrichment analysis included three parts: biological process (BP), cellular component (CC) and molecular function (MF). The top 10 entries of BP, CC, and MF were selected according to *p*-value to draw bar graphs (**Figure 1E**). In addition, KEGG enrichment analysis revealed that these genes may be involved in EGFR tyrosine kinase inhibitor resistance, Rap1 signaling pathway and PI3K/Akt signaling pathway (**Figure 1F**).

### 4.2 ASIV improved neurological function and reduced brain infarction and neuronal apoptosis in MCAO/R mice

**Figure 2:**
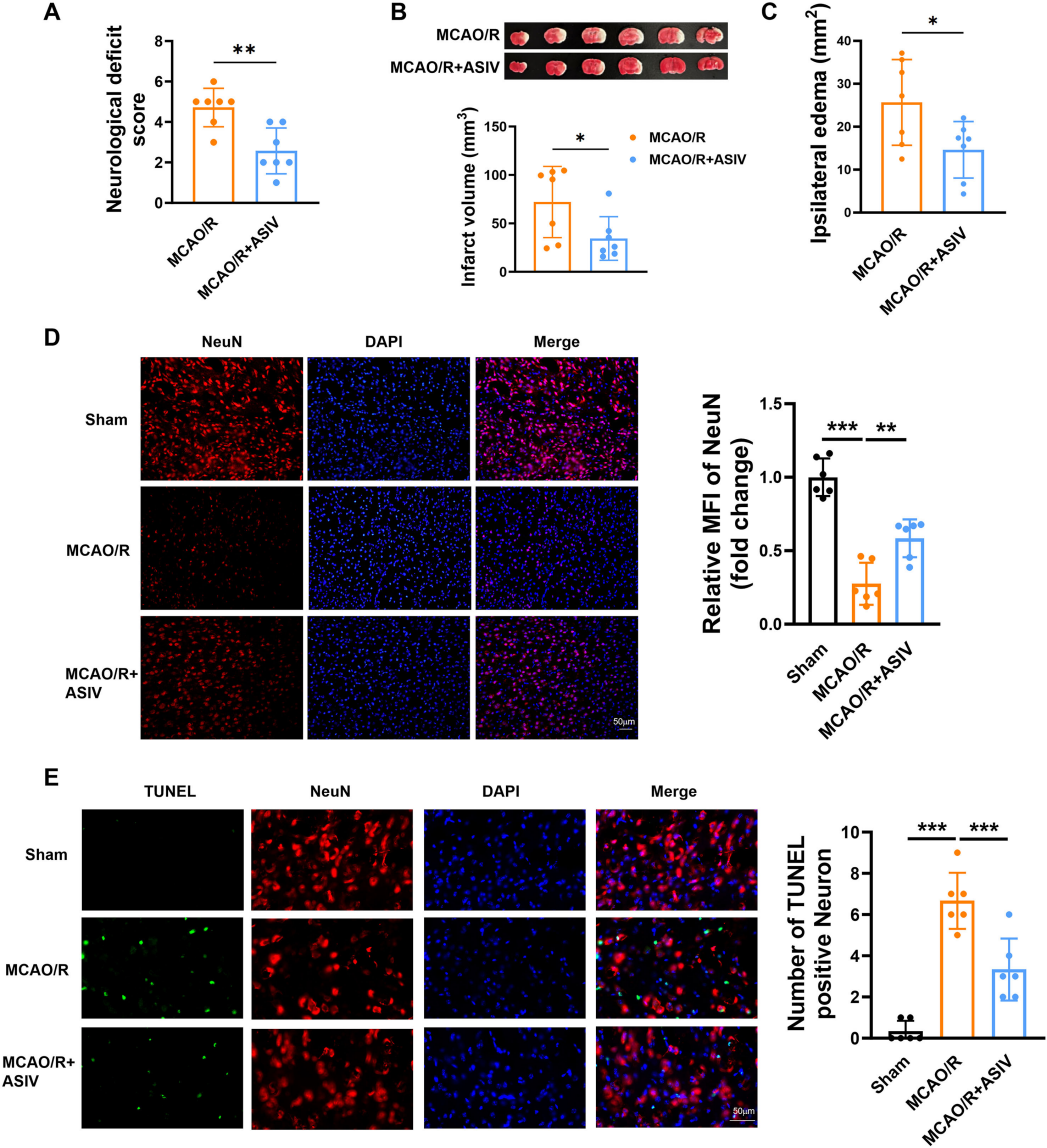
ASIV ameliorated functional deficits and reduced brain infarction and neuronal apoptosis in MCAO/R mice. **(A)** ASIV treatment resulted in a significant reduction in the neurological deficit score of MCAO/R mice. **(B, C)** TTC staining showed ASIV significantly reduced the brain infarct volume and brain edema in MCAO/R mice. **(D)** Immunofluorescence staining showed that ASIV mitigated neuronal loss in the ischemic cortex. **(E)** TUNEL staining revealed that ASIV attenuated neuronal apoptosis in the ischemic cortex (red, NeuN; green, Tunel-positive staining; blue, DAPI core dye). * *p* < 0.05, **p<0.01, ***p<0.001; *n* = 7.

To investigate the therapeutic effects of ASIV on cerebral ischemic injury, a mouse model of MCAO/R was established. Neurological deficit scores revealed severe impairment in the MCAO/R group (4.71 ± 0.36) at 24 h post-modeling. In comparison, ASIV treatment significantly attenuated neurological deficits, reducing scores to 2.57 ± 0.43, indicating substantial functional recovery (**Figure 2A**). TTC staining demonstrated that ASIV administration markedly reduced cerebral infarct volume from 72.05 ± 13.90 mm³ in the model group to 34.41 ± 8.51 mm³ (**Figure 2B**). MCAO/R significantly increased the degree of edema in the ipsilateral brain, while the treatment with ASIV significantly mitigated this effect (**Figure 2C**). Furthermore, MCAO/R induced a significant reduction of NeuN-positive neurons in the ischemic cortex at 24 h post-reperfusion, which was substantially attenuated by ASIV treatment (**Figure 2D**). The neuroprotective effect of ASIV was further corroborated by TUNEL staining (**Figure 2E**). While the MCAO/R group exhibited a substantial increase in neuronal apoptosis in the ischemic cortex compared to the sham group, this elevation was significantly attenuated by ASIV treatment (**Figure 2E**).

### 4.3 ASIV alleviated MCAO/R-induced BBB disruption

**Figure 3:**
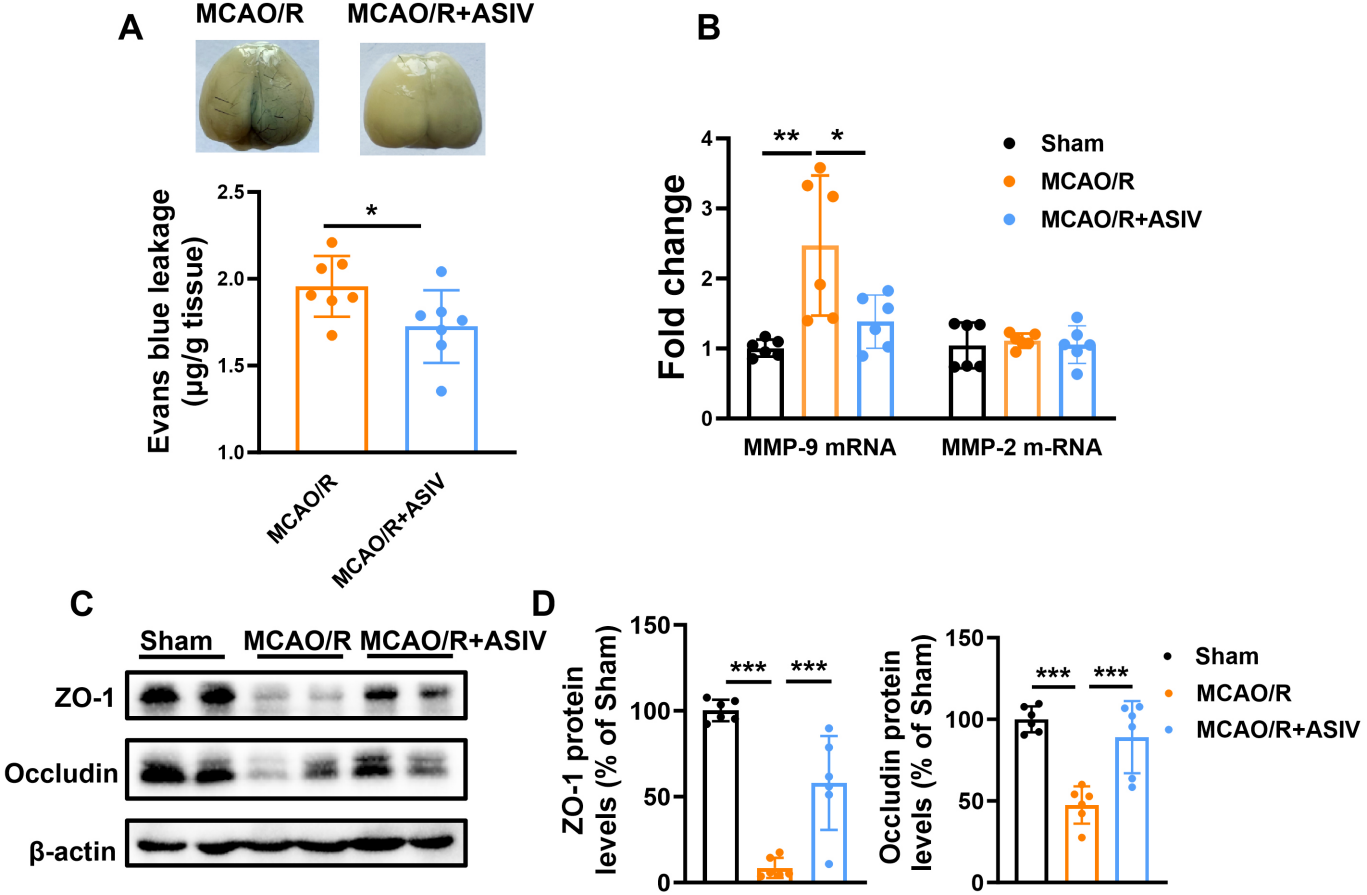
ASIV attenuated MCAO/R-induced BBB disruption, MMP-9 mRNA levels, and tight junction protein degradation. **(A)** ASIV attenuated MCAO/R-induced Evans blue extravasation in the ischemic hemisphere. **(B)** ASIV treatment significantly attenuated the MCAO/R-induced increase in MMP-9 mRNA levels, as determined by RT-PCR. **(C, D)** Western blotting showed that ASIV restored the protein levels of ZO-1 **(C)** and Occludin **(D)** downregulated by MCAO/R. *p < 0.05, **p<0.01, ***p<0.001; n=6.

Evans blue extravasation was employed to evaluate the effect of ASIV on BBB integrity in MCAO/R mice. The MCAO/R group exhibited significantly increased Evans blue extravasation in the ipsilateral hemisphere (1.96 ± 0.06 μg/g), indicating severe BBB disruption. ASIV treatment significantly attenuated this extravasation (1.73 ± 0.08 μg/g), suggesting a protective effect against ischemia-induced BBB damage (**Figure 3A**). Matrix metalloproteinases (MMPs) disrupt the BBB by degrading extracellular matrix and junctional proteins post-ischemia. RT-PCR analysis revealed that the mRNA level of MMP-9 increased over two-fold compared to the sham group, which was significantly attenuated by ASIV treatment (**Figure 3B**). MMP-2 expression showed a decreasing trend with ASIV, though statistically non-significant.

Further investigation was conducted on the effect of ASIV treatment on tight junction proteins (ZO-1 and Occludin) loss in the cerebral microvasculature. The results showed that at 24 h post-MCAO/R, ZO-1 protein levels decreased to 8.53 ± 2.41% of sham group values, while Occludin expression declined to 47.49 ± 4.66%. Compared with the MCAO/R group, ASIV treatment significantly restored tight junction protein expression, elevating ZO-1 to 58.01 ± 11.16% and Occludin to 89.06 ± 9.05% of sham levels (**Figures 3C, D**).

### 4.4 ASIV promoted the phenotype conversion of LPS-insulted BV2 cells from M1 to M2 and modulated the PI3K/Akt/NF-κB signaling in BV2 cells.

**Figure 4:**
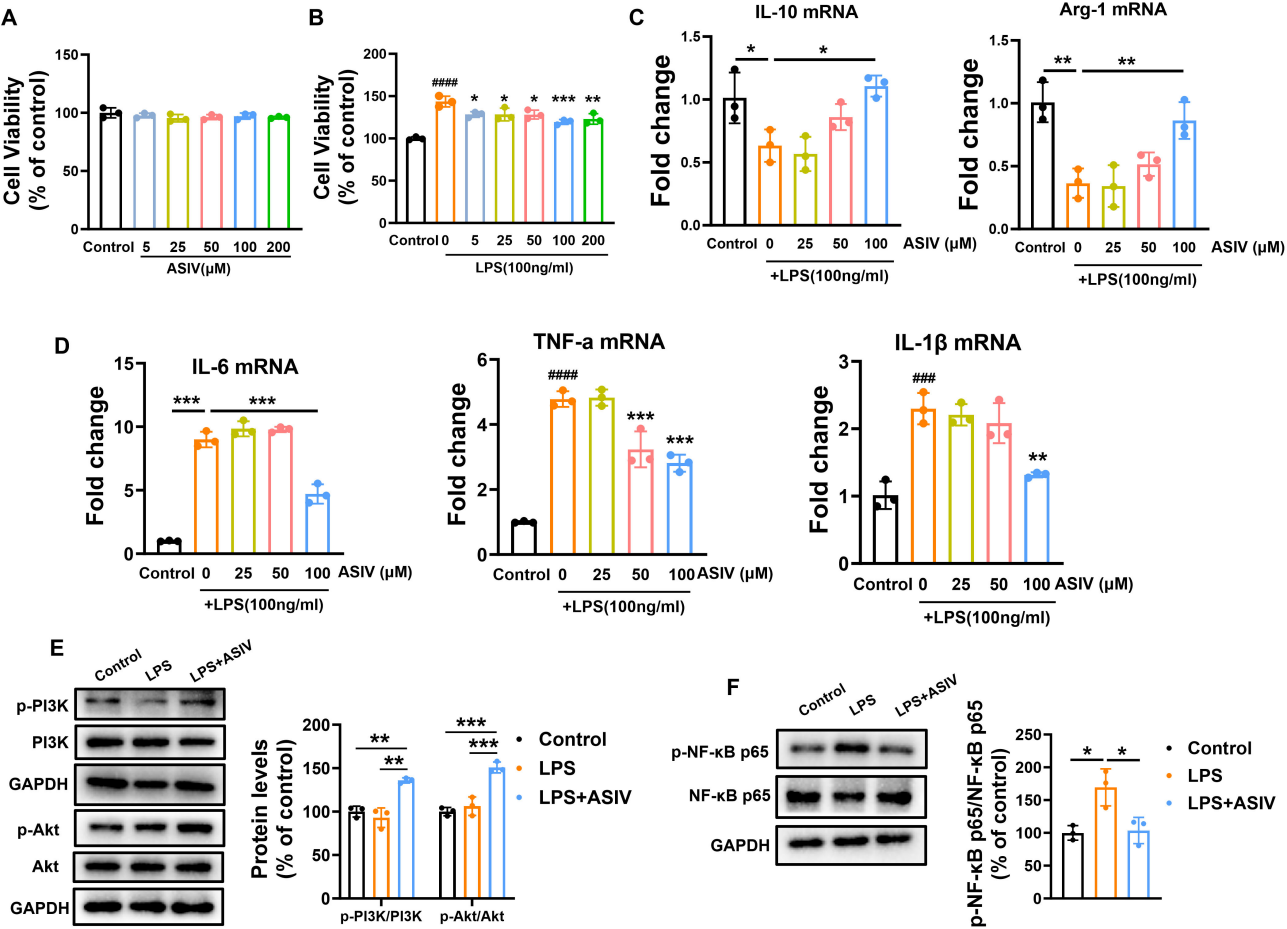
ASIV promoted the phenotype conversion of LPS-insulted BV2 cells from M1 to M2 and modulated the PI3K/Akt/NF-κB signaling in BV2 cells. **(A, B)** The effects of ASIV and LPS on cell viability were assessed by the CCK-8 assay. **(C, D)** The effect of ASIV on the mRNA expression of M1 and M2 markers was assessed by RT-PCR. **(E, F)** Analysis of ASIV effects on LPS-induced PI3K/Akt and NF-κB phosphorylation in BV2 microglial cells by Western blotting. *P < 0.05, **P < 0.01, ***P < 0.001, n=3.

Firstly, the effect of ASIV on BV2 cells survival was investigated, and the results showed that different concentrations (25, 50, 100, and 200 μM) of ASIV had no toxic effects on BV2 cells compared with the control (**Figure 4A**). Subsequently, co-treatment with ASIV and LPS for 24 h significantly suppressed LPS-induced proliferation of BV2 cells across all tested concentrations (**Figure 4B**). Next, the effect of ASIV on the anti-inflammatory M2 markers and pro-inflammatory M1 markers of BV2 cells induced by LPS was evaluated (**Figures 4C, D**). Following 24h LPS exposure, the mRNA levels of anti-inflammatory cytokines (IL-10, Arg-1) were significantly downregulated, while those of pro-inflammatory cytokines (IL-6, TNF-α, and IL-1β) were markedly upregulated. Compared to the control, IL-10 and Arg-1 decreased to 0.63-fold and 0.36-fold, respectively, whereas IL-6, TNF-α, and IL-1βincreased to 8.99-fold, 4.78-fold, and 2.30-fold, respectively. Treatment with ASIV (50 and 100 μM) significantly attenuated the LPS-induced overexpression of TNF-α mRNA, reducing it to 3.23-fold and 2.81-fold of the control level, respectively. Furthermore, 100 μM ASIV not only elevated IL-10 and Arg-1 to 1.11-fold and 0.86-fold, respectively, but also reduced IL-6 and IL-1β mRNA levels to 4.71-fold and 1.32-fold of the control. To further elucidate the mechanism, we investigated whether ASIV modulates LPS-induced phosphorylation of PI3K/Akt and NF-κB (**Figures 4E, F**) in BV2 cells. As shown in **Figure 4E**, LPS stimulation did not significantly alter the phosphorylation levels of PI3K and Akt compared to the control group. However, ASIV treatment markedly increased the phosphorylation of both PI3K and Akt. Furthermore, we observed that ASIV significantly inhibited the phosphorylation of NF-κB in LPS-induced BV2 cells (**Figure 4F**).

### 4.5 ASIV facilitated M2 polarization in LPS-stimulated BV-2 cells via PI3K/Akt/NFκB signaling pathway

**Figure 5:**
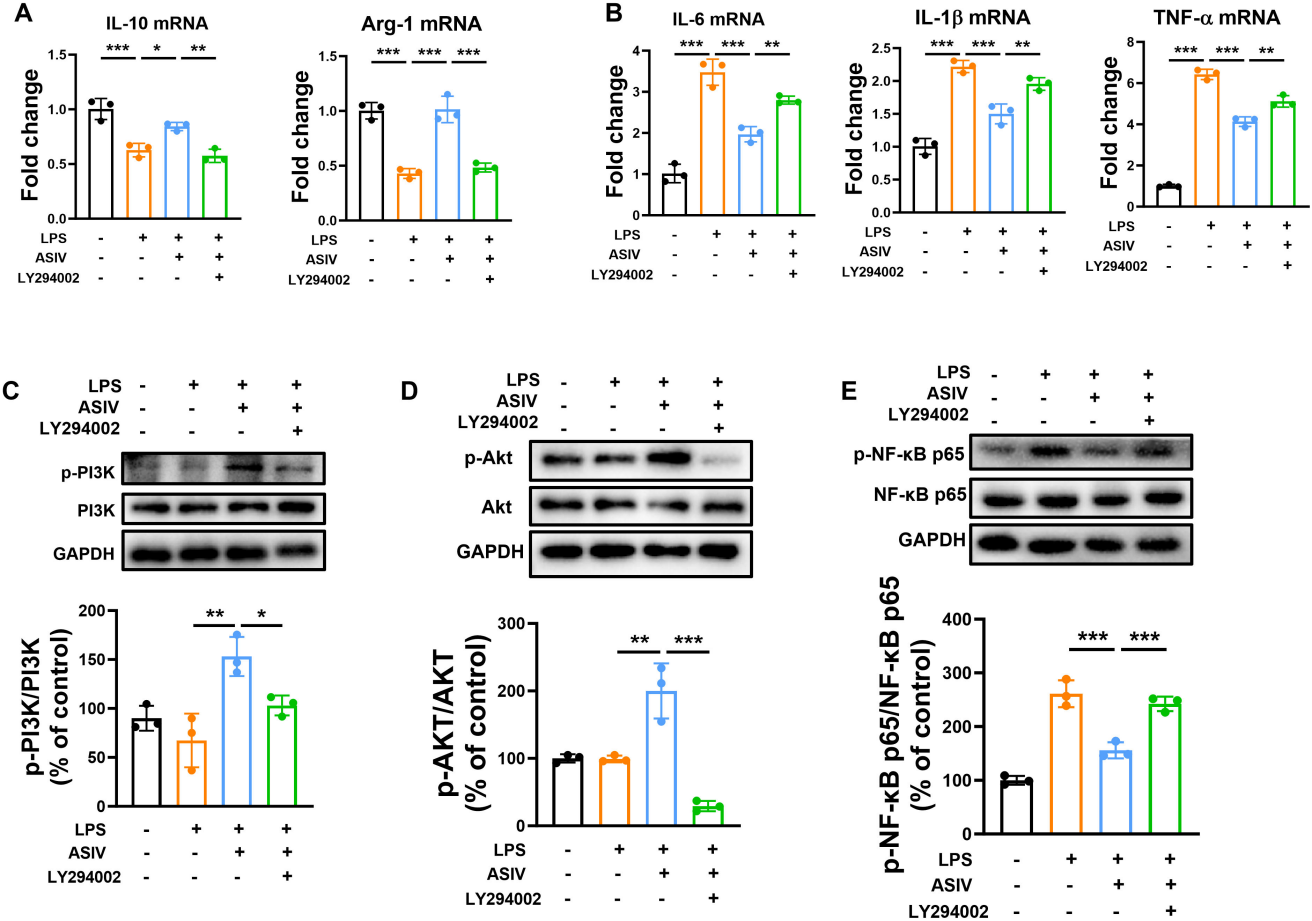
ASIV facilitated M2 polarization in LPS-stimulated BV-2 cells via PI3K/Akt/NFκB signaling pathway. **(A, B)** BV2 cells were pretreated with 10 µM LY294002 (a PI3K inhibitor) for 1 h, followed by LPS stimulation for 24 h. The blocking effect of LY294002 on the ASIV-mediated regulation of M1 and M2 marker mRNA expression was detected by RT-PCR. **(C, D)** As assessed by Western blot, LY294002 blocked the ASIV-induced phosphorylation of PI3K and Akt and reversed the inhibitory effect of ASIV on NF-κB phosphorylation. *P < 0.05, **P < 0.01, ***P < 0.001, n=3.

To further confirm the involvement of the PI3K/Akt signaling pathway, we pre-treated BV2 cells with the PI3K inhibitor LY294002 (10 µM) one hour before LPS induction. As demonstrated in **Figures 5A, B**, ASIV significantly modulated the LPS-induced cytokine expression. It not only potentiated the mRNA expression of anti-inflammatory cytokines (IL10, Arg-1) but also attenuated the expression of pro-inflammatory cytokines (IL-6, IL-1β, and TNF-α). Co-treatment with LY294002 blocked both regulatory effects of ASIV. Furthermore, we investigated whether LY294002 could block the molecular expression changes of the PI3K/Akt/NF-κB signaling pathway. As expected, **Figures 5C–E** showed that LY294002 eliminated the promoting effect of ASIV on PI3K and Akt phosphorylation, and eliminated the inhibitory effect of ASIV on NF-κB phosphorylation. These results indicate that the anti-neuroinflammatory action of ASIV in BV2 cells is dependent on the activation of the PI3K/Akt axis, which subsequently leads to the inhibition of NF-κB activation.

### 4.6 ASIV suppressed neuroinflammation in MCAO/R mice by modulating the PI3K/Akt/NF-κB pathway

**Figure 6:**
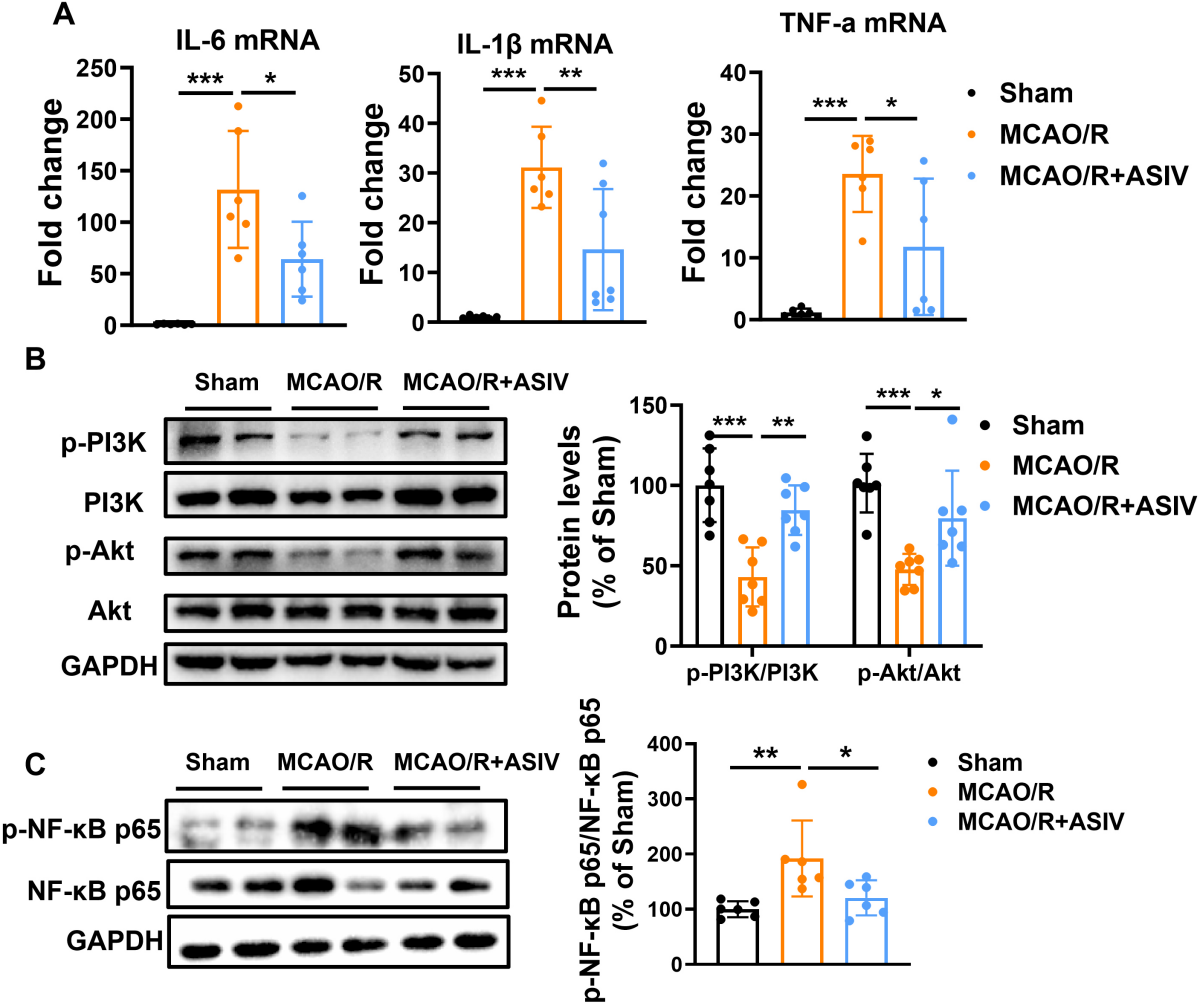
ASIV exerted anti-neuroinflammatory effects on MCAO/R mice through the PI3K/Akt/NF-κB signaling pathway. **(A)** ASIV significantly inhibited the increase of IL-6, IL-1β and TNF-α mRNA expression in the brain of MCAO/R mice as demonstrated by RT-PCR. **(B)** The effect of ASIV on the phosphorylation of PI3K/Akt and NF-κB in the brains of MCAO/R mice was analyzed by Western blot. *P < 0.05, **p < 0.01, ***p < 0.001, n = 6.

**Figure 7:**
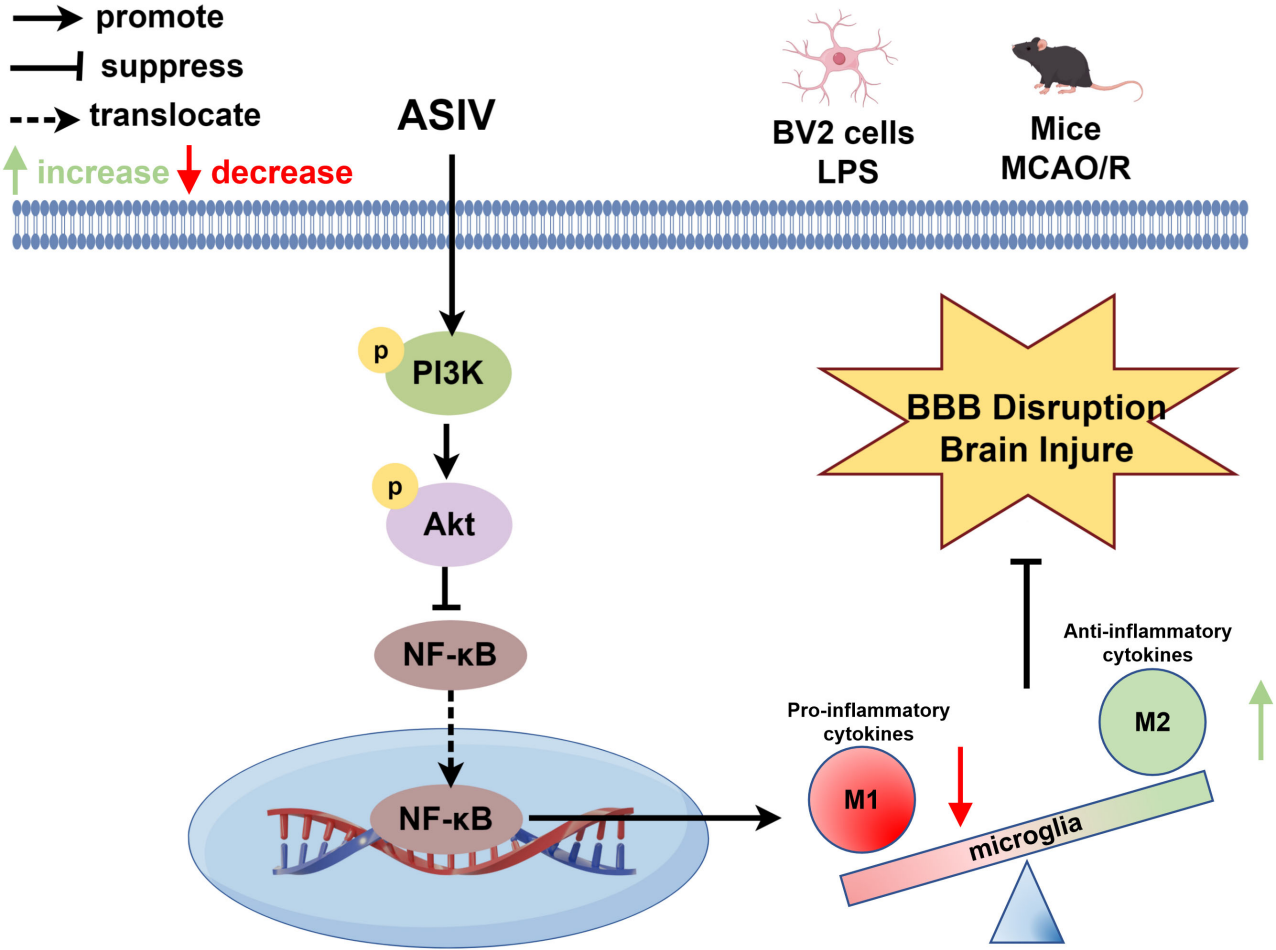
Schematic diagram illustrating the mechanism by which ASIV ameliorates CIRI.

The inhibitory effect of ASIV on inflammatory cytokines was assessed by RT-PCR, and the results showed that the MCAO/R model significantly up-regulated the mRNA levels of IL-6, IL-1β, and TNF-α in the brain tissues compared to the sham-operated group (**Figure 6A**), whereas the ASIV treatment effectively reversed this change. ASIV exerted a sustained regulatory effect on the PI3K/Akt/NF-κB signaling pathway at multiple time points after reperfusion. At 6 h post-reperfusion, MCAO/R markedly suppressed the phosphorylation of PI3K and Akt and activated NF-κB, while ASIV administration rapidly reversed these changes and concurrently inhibited NF-κB activation (**Supplementary Figures 1A, B**). This regulatory effect persisted at 24 h post-reperfusion, where ASIV treatment similarly reversed the MCAO/R-induced suppression of PI3K/Akt phosphorylation and inhibited the activation of NF-κB (**Figures 6B, C**). By 72 h post-reperfusion, phosphorylation of PI3K/Akt remained suppressed in the model group, whereas ASIV continued to enhance their phosphorylation levels and persistently inhibited NF-κB (**Supplementary Figures 1C, D**). These findings indicate that ASIV acts as an early trigger of protective PI3K/Akt signaling and an early suppressor of NF-κB at 6 h, and maintains this sustained modulation through 72 h, supporting its role in multi-phase neuroinflammatory regulation after ischemia-reperfusion. These findings indicate that ASIV acts as an early trigger of protective PI3K/Akt signaling and an early suppressor of NF-κB at 6 h, and maintains this sustained modulation through 72 h, supporting its role in multi-phase neuroinflammatory regulation after ischemia-reperfusion. Collectively, these findings suggest that ASIV plays a role in inhibiting neuroinflammation and maintaining the integrity of the BBB by regulating the PI3K/Akt/NF-κB axis, which ultimately ameliorates CIRI (**Figure 7**).

## 5 Discussion

This study integrated network pharmacology prediction with experimental validation to systematically investigate the therapeutic potential and underlying mechanisms of ASIV in ischemic stroke. Our results demonstrated that ASIV could significantly ameliorate CIRI, as evidenced by the recovery of neurological function and reduction in infarct volume and neuronal apoptosis. More importantly, it played a prominent role in preserving the integrity of BBB and suppressing neuroinflammation. At the mechanistic level, this study found that the activation of the PI3K/Akt signaling pathway and subsequent inhibition of NF-κB signaling pathway are central to mediating these protective effects of ASIV.

Previously, Yu et al. revealed the neuroprotective effects of ASIV in CIRI through network pharmacology and experimental studies, and proposed that it might partially inhibit apoptosis by regulating the JNK/Bid signaling pathway (21). Building upon this foundation, our study conducted a more comprehensive analysis by expanding both drug and disease target databases. We successfully identified a core set of targets, including ALB, TNF, IL6, AKT1, IL1B, MAPK3, EGFR, CASP3, MMP9 and STAT3 (**Figure 1D**). These targets clearly indicate that the mechanism by which ASIV ameliorates CIRI extends beyond solely inhibiting apoptosis to encompass two other critical aspects: suppressing neuroinflammation and maintaining BBB integrity. This computational prediction has clarified a clear direction and focus for our subsequent mechanistic exploration.

Ischemia stroke triggers a complex neuroinflammatory cascade, initiated by microglia transition from a resting to an activated state and releasing abundant pro-inflammatory factors such as IL-1β, IL-6, TNF-α, chemokines, and reactive oxygen species (ROS), subsequently activating astrocytes which further contribute to this process (22–24). Subsequently, various peripheral immune cells, including neutrophils and monocytes/macrophages, are recruited and infiltrate into the brain tissue, drastically amplifying the local inflammatory response (25). Critically, neuroinflammation, as a major driver of BBB disruption, lead to increased BBB permeability, which in turn provides a gateway for the infiltration of peripheral inflammatory cells and the diffusion of inflammatory signals. These processes form a self-reinforcing vicious circle, collectively promoting neuronal death, exacerbating brain edema, and worsening neurological deficits (26–28). The study confirmed that ASIV could significantly inhibit the mRNA expression of key pro-inflammatory cytokines (IL-1β, IL-6, TNF-α) in both brain tissues of MCAO/R mice and LPS-induced BV-2 microglia, demonstrating that ASIV can effectively curb the inflammatory cascade in CIRI. Furthermore, in the LPS-induced BV2 cell model, ASIV effectively increased the expression levels of anti-inflammatory M2 markers (IL-10, Arg-1). Collectively, these results indicate that ASIV promoted the shift of LPS-insulted BV2 cells from an M1 to an M2 phenotype.

The BBB is primarily composed of brain microvascular endothelial cells and their tight junctions, astrocytic end-feet, pericytes, and the basement membrane (26). Neuroinflammation attacks this intricate structure through multiple mechanisms: activated microglia and infiltrating neutrophils secrete large amounts of matrix metalloproteinases (MMPs), particularly MMP-9, which can degrade key components of the BBB basement membran, such as collagen and laminin, and disrupt tight junction proteins, thereby breaking down the structural support of the BBB (29). Pro-inflammatory cytokines (IL-1β, IL-6, TNF-α) can directly downregulate the expression and function of tight junction proteins between endothelial cells, widening the “gaps” and increasing permeability (30). Our study found that ASIV significantly reduced Evans blue extravasation and effectively reversed the ischemia-induced degradation of tight junction proteins (ZO-1 and occludin). Simultaneously, ASIV significantly downregulated the mRNA level of MMP-9. Consistent with this protective effect within the body is the direct functional assessment using brain endothelial cells, which indicates that ASIV significantly maintained the integrity of the barrier under inflammatory challenges. Specifically, Li et al. demonstrated that ASIV prevented the reduction in transepithelial electrical resistance (TEER) induced by LPS, inhibited the increase in intercellular pore permeability, and alleviated the downregulation of tight junction proteins (31). Therefore, ASIV strengthens the structure and function of the BBB through a dual-mechanism: inhibiting MMP-9 expression and stabilizing tight junction proteins.

Based on the KEGG enrichment analysis results, the most significant advancement of this study lies in revealing the core upstream mechanism by which ASIV exerts its protective effects - the activation of the PI3K/Akt signaling pathway. Previous studies have confirmed that in the complex pathological process of CIRI, the PI3K/Akt pathway serves as a pivotal regulatory hub, coordinating multiple cellular responses to counteract damage and promote cell survival (32). Upon activation, the PI3K/Akt pathway primarily mediates neuroprotection through the following mechanisms: inhibiting key pro-inflammatory signaling pathways such as NF-κB, thereby alleviating inflammation-mediated brain tissue damage (33–35); suppressing the activity of pro-apoptotic proteins like Bax and Caspase-3, while upregulating the expression of anti-apoptotic proteins like Bcl-2, thus preventing neuronal apoptosis (36–38); and modulating the level of autophagy to maintain cellular homeostasis (39).

In this study, both *in vivo* and *in vitro* experiments consistently demonstrated that ASIV could significantly enhance the phosphorylation levels of PI3K and Akt. Specifically, ASIV reversed the ischemia-induced inhibition of the PI3K/Akt pathway in the MCAO model and exhibited a potent activating effects on this pathway in LPS-induced BV-2 cells. We further clarified the causal relationship between the PI3K/Akt pathway and anti-inflammatory effect: ASIV inhibits phosphorylation of the NF-κB p65 subunit by activating PI3K/Akt. As the “master switch” of the inflammatory response, NF-κB regulates the transcription of various inflammatory mediators, including IL-1β, IL-6, TNF-α, MMP-9, etc. (40). Additional studies have indicated that NF-κB activation can upregulate the transcription and protein expression of PLA2G4A, thereby promoting the release of inflammatory mediators (41, 42). Zhu et al. found that ASIV exerts neuroprotective effects in CIRI by targeting PLA2G4A through proteomics and network pharmacology analyses (43), a finding which strengthens our conclusions. Notably, beyond the core PI3K/Akt/NF-κB axis validated herein, another key hub target identified in our network, STAT3, has also been experimentally confirmed in our prior study (11). We found that ASIV significantly suppressed STAT3 activation in the ischemic brain, an inhibition that prevented detrimental NK cell responses and improved stroke outcomes. This independent validation of a predicted hub target substantiates the systems-level claim of our network analysis and reinforces the notion that ASIV acts via a concerted mechanism on multiple signaling nodes.

Furthermore, a noteworthy finding is that in BV-2 cells, LPS stimulation itself did not significantly alter the phosphorylation level of PI3K/Akt, whereas ASIV strongly activated this pathway. This suggests that the anti-inflammatory effect of ASIV does not originate solely from antagonism of LPS signaling, but rather from actively enhancing this endogenous pro-survival signaling pathway, thereby creating a cellular environment that is inherently more resistant to inflammatory insults. This provides new evidence supporting its multi-target and holistic regulatory properties. It is also important to acknowledge a potential limitation of this study, which lies in the exclusive use of the BV2 cells line for *in vitro* validation. While BV2 cells are a widely accepted and valuable model for preliminary investigation of microglial responses, they differ from primary microglia in aspects such as inflammatory signaling thresholds and metabolic states. Consequently, the exact magnitude and dynamics of the observed anti-inflammatory effects of ASIV via the PI3K/Akt/NF-κB pathway may not fully recapitulate the response in primary microglia within a more complex physiological or pathological context. Future studies employing primary microglia or human iPSC-derived microglial models would strengthen the physiological relevance and translational potential of these findings.

While our preclinical findings demonstrate the efficacy of ASIV, its potential clinical translation requires careful consideration of key practical barriers. First, regarding the therapeutic time window, our data showing that ASIV modulates the PI3K/Akt/NF-κB pathway and mitigates injury at multiple post-reperfusion time points (6–72 h) suggest a possibly broad intervention window, which is a significant advantage over many neuroprotectants that are effective only within a very narrow timeframe. However, the optimal administration timing relative to reperfusion in a clinical setting warrants further investigation. Second, concerning compatibility with standard reperfusion therapies (e.g., thrombolysis or thrombectomy), ASIV, as a multi-target anti-inflammatory agent, could theoretically serve as an adjuvant to counteract reperfusion-induced inflammatory cascades without interfering with the revascularization process itself. Future studies should explore combination strategies and sequential treatment regimens. Finally, the issue of potential immunosuppressive risks must be acknowledged. Our data indicate that ASIV promotes a shift toward the protective M2 phenotype rather than causing broad immunosuppression, as evidenced by the upregulation of Arg-1 and IL-10. This selective modulation may help maintain host defense while curbing detrimental inflammation, though the overall immune profile in prolonged treatment requires further evaluation. Addressing these translational aspects positions ASIV not merely as a promising candidate but also frames the necessary next steps toward its realistic integration into future stroke therapy paradigms.

## 6 Conclusion

In summary, integrating network pharmacology with experimental validation, this study demonstrates that ASIV protects against CIRI by activating the PI3K/Akt pathway. This activation suppresses NF-κB-driven neuroinflammation and preserves BBB integrity, ultimately mitigating brain damage and improving neurological outcomes. The findings highlight ASIV’s potential as a novel therapeutic agent for ischemic stroke and underscore the importance of targeting the PI3K/Akt/NF-κB pathway for neuroprotection.

## Data Availability

The raw data supporting the conclusions of this article will be made available by the authors, without undue reservation.
