# Supplementary material for: Astragaloside IV suppresses neuroinflammation via PI3K/Akt/NF-κB to ameliorate cerebral ischemia-reperfusion injury based on network pharmacology analysis and experimental validation
Source: Front Immunol. 2026 Apr 2;17:1735000. doi: 10.3389/fimmu.2026.1735000 (PMC13083011; doi:10.3389/fimmu.2026.1735000)
Supplement: Supplementary file 1 [file DataSheet1.docx]

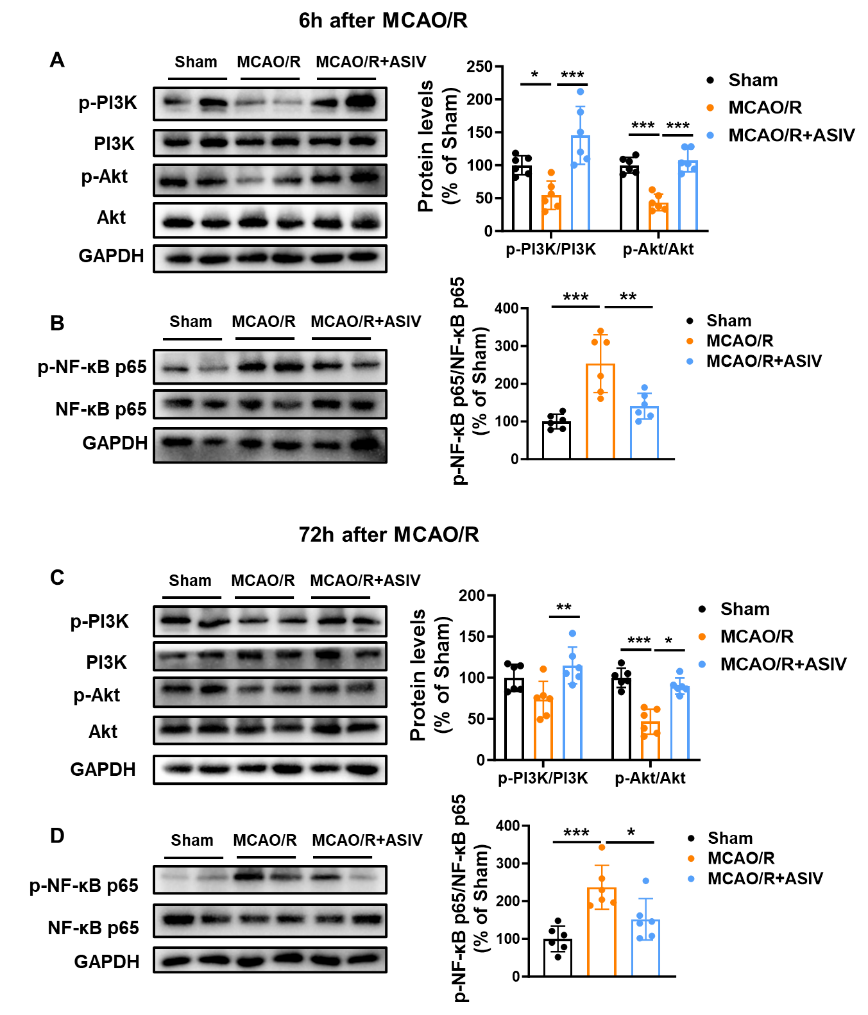


Supplementary Fig. 1 (A-D) Western blot analysis was performed to assess phosphorylation changes in the PI3K/Akt and NF-κB pathways in the brains of MCAO/R mice at 6 and 72 h after reperfusion. **P* < 0.05, ***p* < 0.01, ****p* < 0.001, n = 6.
